# The Potential of the Marine Microalga *Diacronema lutheri* in the Prevention of Obesity and Metabolic Syndrome in High-Fat-Fed Wistar Rats

**DOI:** 10.3390/molecules27134246

**Published:** 2022-06-30

**Authors:** Claire Mayer, Martine Côme, Lionel Ulmann, Isabelle Martin, Graziella Chini Zittelli, Cecilia Faraloni, Khadija Ouguerram, Benoît Chénais, Virginie Mimouni

**Affiliations:** 1 Département Génie Biologique, BiOSSE, Biology of Organisms: Stress, Health, Environment, Institut Universitaire de Technologie, Département Génie Biologique, Le Mans Université, F-53020 Laval, France; claire.mayer@univ-lemans.fr (C.M.); martine.come@univ-lemans.fr (M.C.); lionel.ulmann@univ-lemans.fr (L.U.); isabelle.martin@univ-lemans.fr (I.M.); 2 Department of Biology, Agriculture and Food Sciences, Institute for BioEconomy, National Research Council, Sesto Fiorentino, I-50019 Florence, Italy; graziella.chinizittelli@cnr.it (G.C.Z.); cecilia.faraloni@cnr.it (C.F.); 3 UMR1280 PhAN, Physiopathology of Nutritional Adaptations, INRAe, CHU Hôtel Dieu, IMAD, CRNH Ouest, Nantes Université, F-44000 Nantes, France; khadija.ouguerram@univ-nantes.fr; 4 BiOSSE, Biology of Organisms: Stress, Health, Environment, UFR Sciences et Techniques, Le Mans Université, F-72085 Le Mans, France

**Keywords:** *Diacronema lutheri*, metabolic syndrome, metabolic disturbances, n-3 LC-PUFA, obesity

## Abstract

Long-chain polyunsaturated fatty acids n-3 series (n-3 LC-PUFAs), especially eicosapentaenoic and docosahexaenoic acids, are known to exert preventive effects on obesity and metabolic syndrome. Mainly consumed in the form of fish oil, LC-PUFAs n-3 are also found in significant quantities in other sources such as certain microalgae. The aim of this study was to evaluate the effects of *Diacronema lutheri* (Dia), a microalga rich in n-3 LC-PUFAs, on metabolic disorders associated with obesity. Three groups of male Wistar rats (n = 6 per group) were submitted for eight weeks to a standard diet or high-fat and high-fructose diet (HF), supplemented or not with 12% of Dia (HF-Dia). Compared to HF rats, HF-Dia rats showed a 41% decrease in plasma triacylglycerol (TAG) and an increase in plasma cholesterol (+35%) as well as in high-density lipoprotein cholesterol (+51%) without change to low-density lipoprotein cholesterol levels. Although fasting glycemia did not change, glucose and insulin tolerance tests highlighted an improvement in glucose and insulin homeostasis. Dia supplementation restored body weight and fat mass, and decreased levels of liver TAG (−75%) and cholesterol (−84%). In HF-Dia rats, leptin was decreased (−30%) below the control level corresponding to a reduction of 68% compared to HF rats. Similarly, the anti-inflammatory cytokines interleukin-4 (IL-4) and IL-10 were restored up to control levels, corresponding to a 74% and 58% increase in HF rats, respectively. In contrast, the level of IL-6 remained similar in the HF and HF-Dia groups and about twice that of the control. In conclusion, these results indicated that the *D. lutheri* microalga may be beneficial for the prevention of weight gain and improvement in lipid and glucose homeostasis.

## 1. Introduction

According to the World Health Organization, obesity was defined as an excessive accumulation of fat mass and represents a risk factor for the development of cardiovascular diseases and type 2 diabetes [1]. In forty years, the prevalence of obesity has doubled and one third of the world’s population (approximately 39%) is overweight and/or obese [2]. Among the components of metabolic syndrome, abdominal obesity is characterized by a chronic low-grade systemic inflammation, involving increased plasma pro-inflammatory cytokine levels such as interleukin-6 (IL-6) and tumor necrosis factor-alpha (TNF-α) [3,4]. Insulin resistance associated with metabolic syndrome is responsible for metabolic disturbances such as inflammation, hyperinsulinemia and dyslipidemia [5,6]. Non-alcoholic fatty liver disease (NAFLD) is also a manifestation of the metabolic syndrome and is characterized by an accumulation of neutral lipids in the liver greater than 5.5% [7].

Long-chain polyunsaturated fatty acids n-3 (n-3 LC-PUFAs) showed preventive effects on metabolic syndrome and obesity. Thus, eicosapentaenoic acid (EPA) and docosahexaenoic acid (DHA) exert preventive effects against metabolic disturbances associated with obesity such as dyslipidemia, inflammation, insulin resistance and oxidative stress [8,9,10,11,12,13]. EPA and DHA are mainly found in marine animal sources, including fatty fish such as salmon, tuna and mackerel, and are consumed as fish oil [14,15]. Innovative alternatives to fish oils rich in n-3 LC-PUFAs were developed in the food industry, in particular, contaminant-free microalgal oils [15].

Microalgal oils offer many advantages compared to fish oils, such as being free of an unpleasant smell and less likely to be contaminated with heavy metals. In contrast to fish oils, the controlled cultivation of microalgae avoids variations in their fatty acid composition. Depending on the microalga species, the lipid contents range from 20% to 50% and microalgae are able to accumulate up to 80% of their dry fat mass under stress conditions [16,17]. Moreover, microalgae are a potential source of other highly bioactive molecules such as pigments, phytosterols or proteins [18]. These bioactive molecules are known to exert preventive effects against metabolic disturbances associated with obesity and could be an alternative to fish oils [19]. The Commission Implementing Regulation (EU) 2018/1023 established the list of microalgae authorized as food ingredients such as oil extracted from the microalgae *Ulkenia* sp. and *Odontella aurita*. The oil extracted from *Schizochytrium* sp. is considered a dietary supplement whereas the dried biomass of *Tetraselmis chuii* is classified as specific foodstuffs [20]. Although it can be of interest for human health, *Diacronema lutheri*, a marine microalga rich in n-3 LC-PUFAs and other bioactive molecules such as phytosterols and carotenoids and used in aquaculture, is not yet considered a food ingredient by the Novel Food Regulation of the EU [20,21].

To our knowledge, no study has reported on the effect of *D. lutheri* as a dietary supplement against metabolic disorders associated with obesity. The aim of this study was to evaluate the effects of the marine microalga *D. lutheri*, used as a food supplement, on obesity and metabolic syndrome in high-fat (HF)-fed Wistar rats. Thus, the effect of an HF diet supplemented with 12% of freeze-dried *D. lutheri* during eight weeks was compared to an unsupplemented HF diet in young male Wistar rats. Metabolic and physiological disorders associated with obesity such as being overweight, dyslipidemia, inflammation, hyperleptinemia, insulin resistance, glucose homeostasis and NAFLD were studied. The animal model was a young male Wistar rat, which is commonly used to study metabolic syndrome and obesity. When this model is submitted to an HF diet combined with 10% fructose supplementation in drinking water for eight weeks, it adequately reproduces the different metabolic disturbances encountered in human disease [22,23]. The present work showed the preventive effects of *D. lutheri* against metabolic disorders associated with obesity and metabolic syndrome in young rats submitted to an HF diet.

## 2. Results

### 2.1. Effects of D. lutheri Supplementation on Body Weight and Fat Mass in Wistar Rats Fed a High-Fat Diet

#### 2.1.1. Nutritional Monitoring: Food and Water Intake

**Figure 1:**
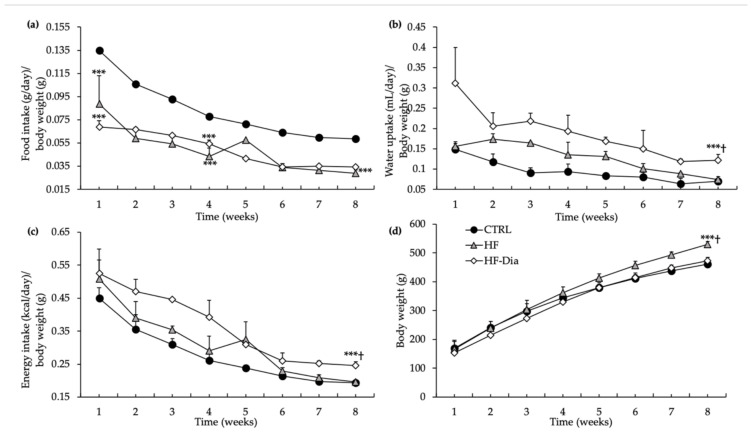
Effect of *D. lutheri* supplementation on food intake (**a**), water intake (**b**), energy intake (**c**) and body weight (**d**). CTRL (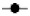), control group; HF (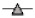), high-fat group; HF-Dia (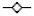), HF supplemented with *D. lutheri* group. Values are means (*n* = 6), with standard deviations represented by vertical bars. Statistical significance was determined using ANOVA with post hoc Fisher’s test and mean values were significantly different from those of the CTRL group with *** *p* < 0.001. ^†^ Significant difference in the HF-Dia group compared with the HF group (*p* < 0.05).

Food, water and energy intake were monitored for eight weeks (Figure 1a–c). The control (CTRL) group displayed the highest food consumption to body weight ratio during the experimental period in comparison with the other groups (Figure 1a). The HF and HF plus *D. lutheri* (HF-Dia) groups presented similar food consumption to body weight ratios during the nutritional protocol. The ratio of water intake to body weight was markedly higher in the HF-Dia group than other groups (Figure 1b). The ratio of water intake to body weight was similar between CTRL and HF rats throughout the protocol (Figure 1b).

#### 2.1.2. Energy Intake

The ratio of energy intake to body weight was similar between CTRL and HF rats except for the first week, where energy intake to body weight ratio was higher in the HF group (Figure 1c). The energy intake to body weight ratio was similar between HF and HF-Dia groups, except for the last week where it was increased in HF-Dia rats compared to the HF group (Figure 1c).

#### 2.1.3. Body Weight and Fat Mass

**Figure 2:**
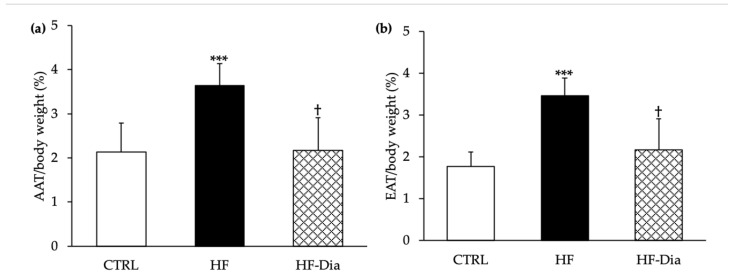
Effects of *D. lutheri* supplementation on abdominal adipose tissue (**a**) and epididymal adipose tissue (**b**) weight. AAT, abdominal adipose tissue; CTRL, control group; EAT, epididymal adipose tissue; HF, high-fat group; HF-Dia, high-fat group supplemented with *D. lutheri*. Values are means (*n* = 6), with standard deviations represented by vertical bars. Statistical significance was determined using ANOVA with post hoc Fisher’s test and mean values were significantly different from those of the CTRL group with ***p < 0.001. ^†^ Significant difference compared with the HF group (*p* < 0.05).

Despite a ratio of energy intake to body weight higher in HF-Dia rats compared to those fed an HF diet at the end of the nutritional protocol, their body weight was significantly different. Indeed, the final body weight was higher in the HF group by comparison with the other groups. The final body weight of HF-Dia rats was similar to that of the CTRL group (Figure 1d). At the same time, abdominal and epididymal adipose tissue weight increased with the HF diet compared to the other groups (Figure 2a,b). Supplementation with *D. lutheri* in HF rats significantly reduced the abdominal as well as epididymal adipose tissue weight/body weight ratios (−40% and −37%, respectively) compared with the HF group, whereas these ratios were similar between the HF-Dia group and CTRL rats (Figure 2a,b).

### 2.2. Proximate Composition, Fatty Acid, Pigment and Sterol Profile of D. lutheri Biomass

#### 2.2.1. Proximate Composition of *D. lutheri* Biomass

As shown in Table S1, protein was the main component (35.4% dry weight (dwt)) of the *D. lutheri* biomass, whereas the lipid content was close to 29% dwt and carbohydrates exhibited the lower value (13.5% dwt). Against a rather low humidity (2.23% dwt), the ash content was high (more than 22% dwt) due to the significant inorganic compounds presented in marine microalgae species. Total dietary fiber (TDF) minerals and the vitamin mixture were not determined in this study.

#### 2.2.2. Fatty Acid Composition of *D. lutheri*

Table S2 shows the fatty acid content and profile of *D. lutheri*. The proportion of total saturated fatty acids (SFAs), monounsaturated fatty acids (MUFAs) and polyunsaturated fatty acids n-3 LC-PUFAs and n-6 LC-PUFAs were also reported. Overall, *D. lutheri* is more abundant in PUFAs than SFAs and MUFAs, in particular in n-3 LC-PUFAs with a n-6 LC-PUFAs/n-3 LC-PUFAs ratio equivalent to 6.15. Moreover, *D. lutheri* showed a very high content of 18:4 n-3 (value greater than 2% dwt) (Table S2). In the same way, 14:0, 16:1 n-7, 18:1 n-9, 18:3 n-3 and 22:6 n-3 fatty acids were found abundantly in the *D. lutheri* biomass (values included between 1 and 2% dwt) (Table S2). In contrast, low levels were observed of 16:2 n-6, 18:2 n-6, 20: 5 n-3, 22:5 n-6 (values included between 0.1 and 1% dwt) and very low levels of 14:1, 15:0, 16:0, 18:3 n-6, 20:0, 20:2 n-6, 20:3 n-3, 20:4 n-6, 22:0, 22:1 and 24:0 fatty acids (values lower than 1% dwt).

#### 2.2.3. Pigment, Antioxidant Activity, In Vitro Digestibility and Sterol Composition of *D. lutheri* Biomass

Pigment composition highlighted high levels in chlorophylls (1.82 dwt), in particular in chlorophyll-a (1.27 dwt) (Table S3). Total carotenoids (TCs) amounted to 0.62 dwt and are responsible for most of the significant antioxidant activity observed. Fucoxanthin was the main carotenoid in *D. lutheri* (54% of TCs), followed by 4k-hex-fucoxanthin (28% of TCs), and to a lesser extent, by dinoxanthin (6.45% of TCs) and β-carotene (2.4% of TCs) (Table S3). As far as the antioxidant properties, the *D. lutheri* biomass exhibited a IC_50_ of 0.33 mg biomass/mL extract for antiradical activity DPPH (2,2-diphenyl-1-picrylhydrazyle) and an ORAC (oxygen radical absorbance capacity) level of 27.4 µmol TE/mg biomass. The “in vitro” dry matter digestibility resulted in about 71% (Table S3), a value slightly lower considering that *D. lutheri* is a naked cell microalga. About sterol composition, Table S3 showed that the main sterols of *D. lutheri* were brassicasterol, stigmasterol and fucosterol (67%, 13.8% and 17% of total sterols, respectively).

### 2.3. Effects of D. lutheri Supplementation on Physiological and Metabolic Disorders in Wistar Rats Fed an HF Diet

#### 2.3.1. Transaminase Levels and Aspartate Amino-Transferase/Alanine Amino-Transferase Ratio

In HF rats, a 17% increase in the alanine amino-transferase (ALAT) plasma level was observed, associated with a decrease in the plasma aspartate amino-transferase (ASAT) level and ASAT/ALAT ratio (−11% and −9%, respectively), compared with CTRL rats (Table 1). Plasma levels of ALAT and ASAT were not restored in the HF-Dia group, but the ASAT/ALAT ratio was lower than those observed in CTRL and HF rats (Table 1).

#### 2.3.2. Plasma Lipids, High-Density Lipoprotein Cholesterol/Low-Density Lipoprotein Cholesterol Ratio and Atherogenic Index of Plasma

The HF group showed a marked increase (+123%) in plasma triacylglycerol (TAG), plasma total cholesterol (TC) and LDL-C levels (+31% and +118%, respectively), and a 7% decrease in plasma HDL-C level in comparison with CTRL rats (Table 1). *D. lutheri* supplementation decreased triglyceridemia (−41%) and increased the plasma TC level (+35%) and HDL-C level (+51%) compared with HF rats (Table 1). However, the LDL-C level remain elevated and was not restored after *D. lutheri* supplementation (Table 1). The atherogenic index of plasma (AIP) was elevated in HF-fed Wistar rats whereas the HDL-C/LDL-C was low compared with the control group (Table 1). Conversely, *D. lutheri* supplementation decreased the AIP (−43%) and increased the HDL-C/LDL-C ratio (+28%) compared with HF rats (Table 1).

### 2.4. Effects of D. lutheri on Glucose Homeostasis and Insulin Sensitivity

#### 2.4.1. Plasma Glucose, Insulin and Leptin Levels and Homeostasis Model Assessment of Insulin Resistance Index

**Figure 3:**
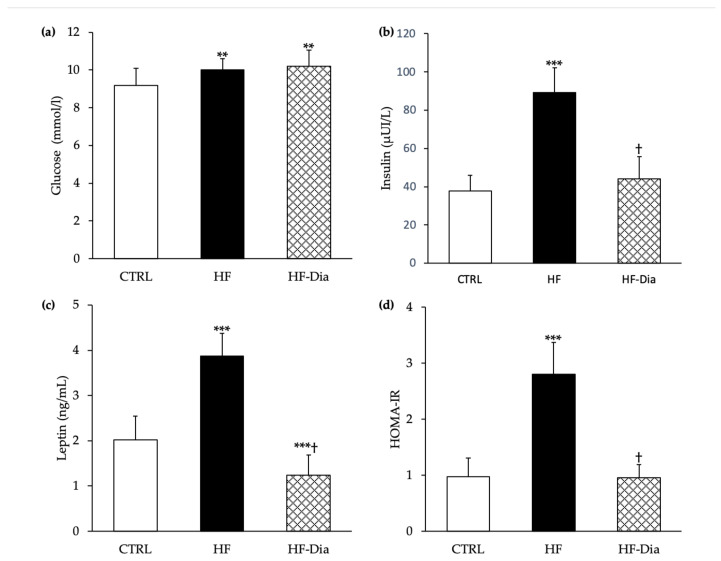
Effects of *D. lutheri* supplementation on plasma glucose (**a**), insulin (**b**) and leptin (**c**) levels and HOMA-IR index (**d**). CTRL, control group; HF, high-fat group; HF-Dia, high-fat group supplemented with *D. lutheri*; TAG, triacylglycerol; TC, total cholesterol. Values are means (n = 6), with standard deviations represented by vertical bars. Statistical significance was determined using ANOVA with post hoc Fisher’s test and mean values were significantly different from those of the CTRL group with ** *p* < 0.01 or *** *p* < 0.001. ^†^ Significant difference compared with the HF group (*p* < 0.05).

In the HF group, basal plasma levels of insulin and leptin were markedly higher compared with the CTRL (+137% and +92%, respectively). In parallel, glycemia was higher in the HF group (+9%) compared with control rats (Figure 3). *D lutheri* supplementation partially prevented hyperinsulinemia observed in the HF group (−50%) and markedly decreased the plasma leptin level (−67%), whereas the glycemia was not restored (Figure 3). In accordance with these results, the homeostasis model assessment of insulin resistance (HOMA-IR) index increased (+188%) with the HF diet compared to CTRL rats and it was restored with *D. lutheri* supplementation (Figure 3).

#### 2.4.2. Glucose and Insulin Tolerance Tests

**Figure 4:**
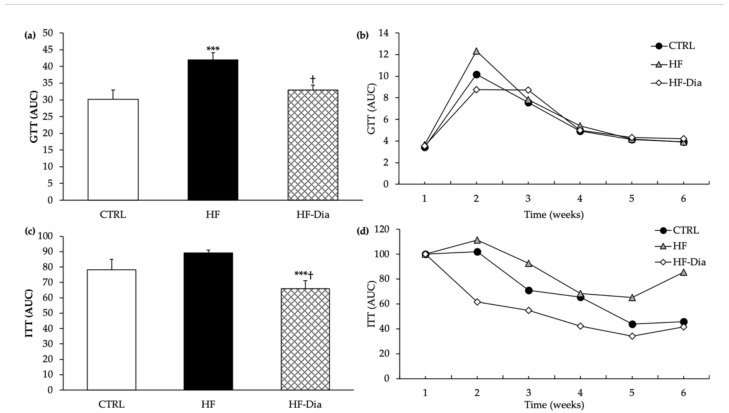
Effect of *D. lutheri* supplementation on glucose homeostasis (**a**,**b**), insulin sensitivity (**c,d**) in HF-fed Wistar rats. CTRL (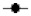), control group; GTT, glucose tolerance test; HF (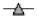), high-fat group; HF-Dia (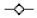), high-fat group supplemented with *D. lutheri*; ITT, insulin tolerance test. Values are means (n = 6), with standard deviations represented by vertical bars. Statistical significance was determined using ANOVA with post hoc Fisher’s test and mean values were significantly different from those of the CTRL group with *** *p* < 0.001. ^†^ Significant difference compared with the HF group (*p* < 0.05).

Glucose and insulin tolerance tests showed beneficial effects of *D. lutheri* in the improvement of glucose homeostasis and insulin sensitivity (Figure 4). An increase in the area under the curve was observed in the HF group compared with the other experimental groups, whereas the HF-Dia group showed a restoration of glucose homeostasis (Figure 4). Although insulin tolerance tests showed no change between control and HF groups, the rats submitted to the HF-Dia diet presented the lowest area under the curve, compared with other experimental groups (Figure 4).

### 2.5. Effects of D. lutheri on Inflammatory Status

As shown in Table 2, plasma concentrations of pro-inflammatory cytokines, including TNF-α and IL-6, were significantly increased in the HF (+86% and +93%, respectively) and HF-Dia groups (+90% and +112%, respectively) compared with those in the CTRL group, suggesting an inflammatory effect of the HF diet. The supplementation with *D. lutheri* did not change the TNF-α level and increased the IL-6 level (+10%) compared to the HF group (Table 2). Anti-inflammatory cytokines were also investigated in plasma and abdominal adipose tissue. The adipose tissue IL-10 concentrations and plasma levels of IL-4 were both decreased by 37% in the HF diet group compared with the CTRL group (Table 2). In contrast, supplementation with *D. lutheri* (HF-Dia) reduced plasma IL-4 and IL-10 concentrations in adipose tissue to the level of the CTRL group (Table 2).

### 2.6. Effects of D. lutheri on Liver Triacylglycerol and Total Cholesterol Levels

**Figure 5:**
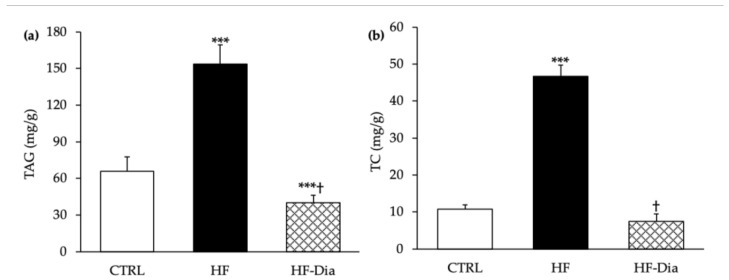
Effects of *D. lutheri* supplementation on liver triglyceride (**a**) and cholesterol (**b**) levels. CTRL, control group; HF, high-fat group; HF-Dia, high-fat group supplemented with *D. lutheri*; TAG, triacylglycerol; TC, total cholesterol. Values are means (n = 6), with standard deviations represented by vertical bars. Statistical significance was determined using ANOVA with post hoc Fisher’s test and mean values were significantly different from those of the CTRL group with *** *p* < 0.001. ^†^ Significant difference compared with the HF group (*p* < 0.05).

The HF diet induced a high increase in hepatic TAG (+136%) and TC contents (+336%) (Figure 5a,b). *D. lutheri* supplementation markedly decreased liver levels of TAG by −74% when compared with HF rats and −39% compared with CTRL rats (Figure 5a), and restored hepatic TC levels (Figure 5b).

## 3. Discussion

The aim of this work was to evaluate the impact of *D. lutheri* used as a food supplement on obesity and associated metabolic disturbances. In accordance with the literature [22,23,24,25,26], our study highlighted that young rats submitted to an HF diet combined with fructose supplementation in drinking water (10%) developed obesity and associated metabolic disturbances, such as increased body weight and fat mass associated with the presence of dyslipidemia, inflammation, hepatotoxicity and NAFLD. In addition, glucose homeostasis was impaired in association with hyperglycemia, hyperinsulinemia, hyperleptinemia and a high AIP. However, insulin sensitivity was not impacted in HF-fed Wistar rats. Indeed, according to the literature, impaired insulin sensitivity is a more variable and non-systematic physiological disturbance in the study of metabolic syndrome in Wistar rats [24]. Our results showed that *D. lutheri* supplementation induced body weight and fat mass reduction. It contributed to an improvement in insulin sensitivity, glucose homeostasis and plasma lipid levels associated with a significant reduction in leptinemia, the AIP and liver lipid levels. The preventive effects of *D. lutheri* on obesity and associated metabolic disorders could be explained by the synergistic effects of various bioactive molecules, including n-3 LC-PUFAs, pigments and phytosterols.

### 3.1. Nutritional Profile of D. lutheri Biomass

The dry biomass of *D. lutheri* has been poorly studied thus far, but it deserves attention as it combines medium-high levels of protein [27] and carbohydrates [28,29] with high lipid content and both EPA and DHA in significant amounts [30], according to the literature. Furthermore, it is well known that D. lutheri, being small in size and lacking a structured cell wall (naked), is expected to be readily digested [31]. All these attributes make it a potential candidate ingredient for diets aimed to exert preventive effects on obesity and metabolic syndrome. Encouraging results in this direction have been recently observed by our group [32] using a diet supplemented with 12% freeze-dried P. tricornutum, a marine microalga rich in carotenoids, especially fucoxanthin, and EPA.

With regard to the major fatty acids, saturated fatty acids 14:0 (1.9%), monounsaturated fatty acids 16:1 n-7 (1.5%) and 18:1 n-9 (1.8%), n-3 polyunsaturated fatty acids 18:4 n-3 (2.3%) and DHA (1.3%) were found. All these five fatty acids accounted for more than 71% of the total fatty acids, with DHA representing 11% of the total fatty acid content whereas the proportion of EPA was less than 2% of total fatty acids. Our results disagree with several reports showing EPA content higher than the DHA level in D. lutheri. However, it is essential to point out that biochemical composition of microalgae is significantly affected by culture conditions (temperature, salinity, light, pH and nutrients, as well as culture regimen) that allows modulation of cell growth and the synthesis and accumulation of high-value products such as PUFAs [33,34].

Among carotenoids, fucoxanthin represents more than 50% of total carotenoids and its importance is due to its bioactivity and health benefits [35]. The antioxidant capacity in the species studied here was higher than the ones reported previously by Ahmed et al., 2014 [36], confirming the suitability of D. lutheri to exert a great antioxidant activity.

Plant sterols are well-known for their ability to reduce LDL-C and promote cardiovascular health, as well as anti-inflammatory, anti-atherogenicity and anti-oxidative activities [18,37]. Considerable amounts of phytosterols are present in microalgae and *D. lutheri* is considered a high-level producer of phytosterols [38]. The sterol pattern of the *D. lutheri* biomass used in the present study contained seven different sterols, of which three (brassicasterol, stigmasterol and fucosterol) represented more than 97% of the total sterols detected (Table S3).

Standards for the evaluation of nutritional value of microalgae include the assessment of various parameters including digestibility [39]. In vitro digestibility provides useful information about the nutrient bioavailability of a product. Most of the literature on algae deals with macroalgae and only a few studies, to our knowledge, focus on the in vitro digestibility of microalgae [28,40]. In the present study, in vitro digestibility obtained in *D. lutheri* (about 71%) was similar to the values obtained by Niccolai et al., 2019 [28] for marine species, although they were lower than expected for a naked cell microalga; it is very likely some polysaccharides produced during growth can create a kind of adhesive film that prevents biomass dispersion, favoring clump formation and, therefore, reducing cell access to proteolytic enzymes.

### 3.2. D. lutheri Supplementation Reduced Body Weight, Abdominal and Epididymal Adipose Weights in HF-Fed Wistar Rats

Similar food and energy intake were observed between the HF and HF-Dia groups, with the exception of the last week of the nutritional protocol. In parallel, the body weight of the HF-Dia rat group was restored compared with the HF one. This result suggests that the decrease in body weight observed in HF-Dia rats is not caused by a deficit in energy intake and/or a decrease in food intake, but may be due to the nutritional quality provided by the *D. lutheri* biomass.

In the present study, the high levels of DHA in the *D. lutheri* biomass (1.33% of dry weight, equivalent to 24 mg DHA/rat/day for the HF-Dia group) could explain the restoration of body weight and fat mass observed in the HF-Dia group. Indeed, previous studies conducted on animals showed the beneficial effects of DHA in reducing hypertrophy and hyperplasia of fat cells by activation of transcription factors involved in preadipocyte differentiation such as peroxisome proliferator-activated receptor gamma and the inhibition of mitogen-activated protein kinases involved in the last phase of adipocyte differentiation [41,42,43]. The study of Kim et al. [42] showed that DHA induces apoptosis of adipocytes before the differentiation stage and reduces lipid accumulation in adipocytes and the number of lipid droplets through increased lipolysis associated with activation of peroxisome proliferator-activated receptor gamma coactivator-1 alpha (PPAR-α), as well as induction of uncoupling protein-2. Furthermore, it was shown that the anti-obesogenic effect of DHA would be mediated by overexpression of enzymes involved in the hydrolysis of di- and triglycerides in adipose tissue such as the hormone sensitive lipase and triglyceride hydrolase [44].

Furthermore, high levels of fucoxanthin in the *D. lutheri* biomass (53.5% of carotenoids, 7.8% of total pigments and 0.33% of dry weight, equivalent to 6 mg fucoxanthin/rat/day for the HF-Dia group) could be another explanation for the decreased body weight and fat mass observed here in HF-Dia rats. In agreement with the present study, similar effects were observed in mice supplemented with a lipid fraction rich in fucoxanthin (9.6% of dry weight) from the macroalga *Undaria pinnatifida* [45]. Another study demonstrated that fucoxanthin could stimulate energy expenditures and β-oxidation associated with a decrease in adipogenesis, through the regulation of PPAR-α, PPAR-γ and uncoupling protein-1 gene expression, leading to a loss of body weight and fat mass [46]. A previous animal study demonstrated that chlorophyll supplementation (0.18 mg/10 g body weight/day) could also exert beneficial effects against obesity by a decrease in body weight gain, an improvement in glucose tolerance and a reduction in low-grade inflammation in HF-fed C57BL/6 J male mice [47]. These effects could be due to the preventive action of chlorophyll supplementation on gut dysbiosis, characterized by the decreased *Firmicutes* to *Bacteroidetes* ratio, the increased abundance of *Blautia* bacteria, and the significant decrease in *Lactococcus* and *Lactobacillus* bacteria [47].

According to the study of Jung et al. [48], fucosterol, a phytosterol from the macroalga *Ecklonia stolonifera*, decreased the accumulation of 3T3-L1 preadipocytes via the inhibition of transcription factors PPARγ and CCAAT/enhancer binding protein alpha [48]. Thus, fucosterol, which is also abundant in the *D. lutheri* biomass (17% of total sterols, 0.178% of dry weight), could exert anti-obesogenic effects in rats supplemented with *D. lutheri*.

### 3.3. Effects of D. lutheri as a Dietary Supplement in the Prevention of Dyslipidemia and Atherosclerosis

In our present work, *D. lutheri* showed beneficial effects against dyslipidemia induced by an HF diet, as evidenced by the improved plasma TAG level and elevated plasma HDL-C level. Some microalgae are known to exert positive effects in the regulation of lipid metabolism [49,50,51]. Among them, *D. lutheri* is distinguished by its richness in phytosterols (1.05% of the microalgal dry weight), which is equivalent to 18.9 mg/HF-Dia rat. In addition, fucosterol exerts a strong hypocholesterolemic activity as shown by its ability to increase plasma HDL-C levels through the activation of the transcription factor liver X receptor [52].

The decrease in plasma TAG levels observed here in HF-Dia rats could be explained by the hypotriglyceridemic properties of n-3 LC-PUFAs, previously found in the *D. lutheri* biomass, via the inhibition of triglyceride production and hepatic lipogenesis [12]. In the same way, a decreased plasma TAG level was previously shown in an HF-fed Wistar rats’ diet supplemented with 0.5% fish oil rich in n-3 LC-PUFAs [53].

An increase in total cholesterol and LDL-C levels was observed in *D. lutheri*-supplemented rats, suggesting, consistent with previous studies, the ability of certain carotenoids such as fucoxanthin to increase plasma cholesterol levels in rodents [54,55].

Dyslipidemia is a major risk factor for atherosclerosis and the increase in the AIP is a good predictor of atherosclerosis, as well as an index of abdominal obesity [56,57]. In the present study, rats supplemented with *D. lutheri* showed a significant decrease in the AIP compared to other experimental groups, which could be explained by cardioprotective effects of the biomolecules found in *D. lutheri* such as n-3 LC-PUFAs and phytosterols [58,59,60,61].

### 3.4. Effects of D. lutheri Supplementation on Inflammatory Status in HF-Fed Wistar Rats

In this work, plasma and adipocyte levels of anti-inflammatory cytokines (IL-4 and IL-10) were restored in HF-Dia rats and these data reflected the partial restoration of basal inflammatory status in rats supplemented with *D. lutheri*, suggesting that DHA from *D. lutheri*, known for its anti-inflammatory effects, increased anti-inflammatory cytokine production [62]. Furthermore, similar effects were observed in a previous animal study that used DHA-rich oil from the microalga *Schizochytrium* sp. as a dietary supplement in C57BL/6 mice submitted to an HF diet [63]. Moreover, microalgal carotenoids also showed anti-inflammatory properties, in particular by increasing anti-inflammatory cytokine levels in different experimental animal models [64]. Unexpectedly, an increased IL-6 plasma level was observed in HF-Dia rats compared to the HF group. This result could be related to the increase in transaminases observed in HF-Dia rats. Indeed, increased levels of IL-6, TNF-α and ALAT are associated with acute liver injury in rats [65,66].

In agreement with our results, inflammation is known to lead to hyperleptinemia, a marker of the pro-inflammatory state, which is positively correlated with fat mass [67,68]. The *D. lutheri* supplementation decreased significantly the plasma leptin level in HF-fed Wistar rats compared with other groups. The study conducted by Yu et al. [63] also showed a leptinemia decrease after eight weeks of treatment in HF-fed C57BL/6 J mice supplemented with *Aurantiochytrium* microalga oil rich in DHA. Moreover, fucoxanthin could be involved in leptinemia regulation in HF-Dia rats. Indeed, a previous study showed the decrease in leptinemia in C57BL/6 J mice submitted for eight weeks to an HF diet combined with *P. tricornutum* extract rich in fucoxanthin (corresponding to 0.2% fucoxanthin) [69]. In this context, high plasma leptin levels are positively correlated with insulin resistance [70]. Thus, to investigate this point, the effects of *D. lutheri* on insulin sensitivity and glucose homeostasis were evaluated.

### 3.5. Effects of D. lutheri Supplementation on Glycemia and Insulinemia and Its Impact on Glucose Homeostasis and Insulin Sensitivity in HF-Fed Wistar Rats

In this study, supplementation with *D. lutheri* restored the basal glucose homeostasis whereas glycemia was not impacted in HF-fed Wistar rats. At the same time, our results highlighted the restoration of insulinemia in the HF-Dia group. Although insulin resistance was not observed in HF rats, insulin tolerance tests (ITT) showed that *D. lutheri* supplementation improved insulin sensitivity. Thus, *D. lutheri* exerts here a preventive role against hyperinsulinemia, suggesting an anti-diabetic activity as observed with various species of microalgae such as *Nannochloropsis oculata*, *Isochrysis galbana*, *Chlorella vulgaris*, *Chlorella pyrenoidosa*, *C. sorokiniana* and *Parachlorella beijerinckii* [71,72,73,74].

The study of Yook et al. [75] showed preventive effects against hyperinsulinemia in C57BL/6J mice submitted to the HF diet supplemented with oil of *Aurantiochytrium* sp., suggesting the beneficial effect of n-3 LC-PUFAs, in particular DHA, in improving insulin sensitivity. Moreover, lipid extract rich in fucoxanthin, derived from the macroalga *Undaria pinnatifida*, showed beneficial effects in the reduction of plasma glucose and insulin levels in obese and diabetic mice [76]. This suggested that fucoxanthin could improve insulin sensitivity and glucose homeostasis through the regulation of the glucose transporter and the decrease in hyperinsulinemia and gluconeogenesis, and impairment of the enzymatic activity of hepatic glucose regulatory enzymes [77].

### 3.6. Effects of D. lutheri Supplementation on Non-Alcoholic Fatty Liver Disease

In vivo, a strong decrease in the liver TAG level was observed in our work in rats from the HF-Dia group, suggesting a preventive effect of *D. lutheri* as a dietary supplement in the prevention of NAFLD. A similar effect was observed in Wistar rats supplemented with the marine microalga *Diacronema vlkianum* for 66 days (equivalent to 101 mg/kg EPA and DHA in the diet), suggesting the preventive effect of n-3 LC-PUFAs in the development of NAFLD [78].

In HF-fed C57BL/6 mice, a protective effect of fucoxanthin against liver lipid accumulation was demonstrated via the reduction in hepatic lipogenic enzymes such as glucose-6-phosphate dehydrogenase, fatty acid synthase and phosphatidate phosphatase, as well as promotion of enzymes involved in β-oxidation [79].

The phytosterol content also plays a beneficial role in the reduction of liver lipid accumulation and could explain the low liver TAG level observed in HF-Dia rats. Indeed, the fucosterol present in the *D. lutheri* biomass could decrease lipid accumulation in the liver by promoting hyperplasia of lipid droplets and decreasing its hypertrophy [80]. Fucosterol is an agonist of liver X receptor transcription factor, which modulates the expression of genes involved in cholesterol homeostasis without leading to liver TAG accumulation [81].

Altogether, these results highlight the potential of the *D. lutheri* biomass and its biomolecules to be exploited in human nutrition [82], especially to prevent metabolic syndrome. However, some data suggest hepatotoxicity in rats supplemented with *D. lutheri* at a dose of 12%, as indicated by the ASAT/ALAT ratio, which was significantly lower in the HF-Dia group due to the higher plasma ALAT level. This potential hepatoxicity of *D. lutheri*, which is the main limitation of our study, deserves further evaluation, including histopathological assessment, and dose-response and time-dependent studies. Thus, determining the safety dose of *D. lutheri* to be incorporated in an HF diet should be the priority for future studies.

## 4. Materials and Methods

### 4.1. Animal and Diets

Eighteen young male Wistar rats, aged three weeks, weighing about 130 g and obtained from Janvier Labs (Le Genest-Saint-Isle, France), were selected to avoid age effects on metabolic disorders associated with obesity, and to avoid any sexual endocrine fluctuation. The nutritional protocol was previously described [32,83] and all the experiments were approved by the Ethical Committee 06 Pays de la Loire and by the French Ministry of Higher Education, Research and Innovation (APAFIS 10187, 31 August 2017).

The animals were randomly divided into three groups of six rats and received diets ad libitum for eight weeks as follows: (1) the CTRL group; (2) the HF group was fed the 260 HF diet (Safe, Augy, France) with 10% fructose in ad libitum drinking tap water (Distriborg, St.-Genis-Laval, France); (3) the HF-Dia group received an HF diet supplemented with 12% (w/w) of the freeze-dried microalga *D. lutheri* (IBE-CNR, Florence, Italy). The amount of *D. lutheri* added to the diet was chosen from our previous animal studies that showed the anti-obesogenic effects of *O. aurita*, *P. tricornutum* and *T. lutea* marine microalgae at 12% (w/w) after eight weeks of the diet in Wistar rats [32,53,83]. These microalgae, which are rich in n-3 LC-PUFAs, showed preventive effects on obesity and metabolic disturbances associated in HF-fed Wistar rats [32,53,83]. Microalgal supplementation was incorporated directly in the HF diet to create a homogeneous mixture and provide 5.93 kcal/g in the HF-Dia diet. DHA content in *D. lutheri* was 1.33% of dry matter, equivalent to an average DHA intake of 24 mg/day/rat.

The daily food, water consumption, energy intake and body weight were monitored as previously described [32,83].

### 4.2. D. lutheri Biomass Preparation and Characterization

The 601 *D. lutheri* microalgae were purchased on 23 July 2015 from the Algal Collection of the University of Naples Federico II (ACUF, http://www.acuf.net/, last accessed on 16 June 2022), Naples, Italy. The biomass used in feeding trials was produced under a greenhouse in closed photobioreactors (from 100 to 500 l working volume), adopting a weekly batch regimen. At harvesting time, cultures were centrifuged to obtain a paste of about 80% moisture that was then frozen at −20 °C until time for the freeze-drying process. Frozen samples were lyophilised under a vacuum at −40 °C and dried material was then finely ground and the powder stored under a vacuum at 20 °C.

In Table S1, the main components of the CTRL and HF diets were presented (SAFE, Augy, France). Proteins, carbohydrates, lipids, ashes and moistures of *D. lutheri* biomass were analyzed (Table S1). Total protein content was estimated as N × 6.25, where N is the nitrogen content determined through the elemental analysis. Carbohydrates were measured following Dubois et al. [84]. Moisture and ashes were determined following ISTISAN protocols (ISTISAN Report 1996/34, method B, p. 7; ISTISAN Report 1996/34, pp. 77–78, respectively). Lipids of *D. lutheri* biomass were determined according to Marsh and Weinstein [85] (Table S1) and the fatty acid composition was reported in Table S2, according to the ISO 12966-4:2015 + ISO 12966-2:2011 procedures. Pigment and sterol compositions, in vitro digestibility and antioxidant activity of *D. lutheri* were reported in Table S3. Pigment composition was determined by the SCOR-UNESCO method [86]. Determination of carotenoid composition was performed by HPLC analysis according to Van Heukelem and Thomas [87]. In vitro digestibility was evaluated by the method of Boisen and Fernández [88], modified as reported by Batista et al. [89]. Antioxidant activity of extracts in 90% acetone was measured by using the DPPH radical scavenger according to the method of Bondet et al. [90] with slight adaptations, reported in Table S3.

### 4.3. Gucose and Insulin Tolerance Tests

To avoid interferences of the ITT and GTT on biochemical parameters, the ITT and GTT were performed on the seventh week of protocol. All rats were submitted to the ITT and GTT (n = 6), with a 3 day recovery between them. Rats were fasted for 6 h before i.p. injection of insulin (1.2 mU/g). Before intraperitoneal (i.p.) injection of glucose (2 mg/g weight), rats were fasted for 12 hrs. From the tail vein of rats, glycemia was monitored with a commercial glucometer (Abbott Diabetes Care Inc., Alameda, CA, USA) for 2 h at the 15th, 30th, 60th and 90th min of injection. The data were expressed as the area under the curve (AUC) in a percentage of glycemia.

### 4.4. Blood and Organ Sampling

After the 8-week nutritional protocol, all rats were fasted for 12 h and anesthetized by intraperitoneal administration of a diazepam–ketamine mixture (4:3, *v/v*). Blood was collected from the abdominal aorta and sampled in 10% ethylenediaminetetraacetic acid (EDTA)-coated tubes (from Sigma, St. Louis, MO, USA). Whole blood was centrifuged at 1000× *g* for 10 min; the supernatant containing the plasma fraction was aliquoted into polyethylene tubes and the red blood cell (RBC) pellet was collected separately. Then, the plasma and RBC samples were stored at −20 °C. Liver, epididymal and abdominal adipose tissues were collected, rinsed with ice-cold NaCl solution (0.9%), weighed, frozen in liquid nitrogen and stored at −80 °C until analysis.

### 4.5. Biochemical Analyses and Hepatic Lipid Measurements

Plasma levels of TAG, TC, HDL-C, ASAT and ALAT, as well as hepatic TC and TAG levels, were measured by enzymatic methods using commercial enzyme kits (BIOLABO, Maizy, France). The ASAT/ALAT ratio was calculated from ASAT and ALAT measurements. The atherogenic risk was evaluated according to Frohlich et al. [91]. Plasma LDL-C levels were estimated from the difference between TC and HDL-C. The HDL-C/LDL ratio was also estimated. Insulin and leptin levels as well as plasma pro-inflammatory cytokines including interleukin-6 (IL-6), tumor necrosis factor-alpha (TNF-α), plasma and adipose anti-inflammatory cytokines such as interleukin-4 (IL-4) and interleukin-10 (IL-10) were quantified as previously described [32,83]. The HOMA-IR index was estimated according to Matthews et al. [92].

### 4.6. Stastistical Analysis

Data from experimental analyses were presented as mean values ± standard deviation (SD) (*n* = 6). After the analysis of variance by one-way ANOVA, the mean values were compared using Fisher’s least significant difference post hoc test (LSD). All statistical analyses were performed with Statgraphics Plus 5.1 (Manugistics Inc., Rockville, MD, USA).

## 5. Conclusions

This study demonstrated the beneficial effects of the marine microalga *D. lutheri* as a dietary supplement in the prevention of obesity and associated metabolic disorders in HF-fed Wistar rats. Our results showed that *D. lutheri* has the potential to prevent abdominal obesity, hyperinsulinemia, hyperleptinemia, hypertriglyceridemia and NAFLD, as well as to improve glucose homeostasis and insulin sensitivity. Further studies are needed to attribute the observed effects to any of the microalgal bioactive molecules (i.e., n-3 PUFAs, fucoxanthin, phytosterols, fibers, etc.) and their potential synergy within the whole *D. lutheri* biomass. However, an increase in plasma transaminase levels (particularly ALAT) was observed in *D. lutheri* supplemented rats, suggesting hepatoxicity at the 12% dose. Therefore, a new animal study with a lower dose of *D. lutheri* supplementation should be performed to support its potential and promising use as dietary supplement in human nutrition. Once the safety dose is defined, a mechanistic approach could be interesting to study the activation of the insulin receptor pathway and lipid metabolism in liver and adipose tissue. Similarly, gene and protein expression of inflammation-related indicators and molecular signaling pathways related to glycolipid metabolism could be explored in the future.

## Figures and Tables

**Table 1:** Effects of *D. lutheri* on plasma biochemical parameters.

Plasma Biochemical Parameters	CTRL	HF	HF-Dia
ASAT (UI/L)	58.72 ± 6.22	51.80 *** ± 7.83	52.53 *** ± 5.06
ALAT (UI/L)	44.46 ± 4.57	52.10 *** ± 4.10	56.29 ***^†^ ± 4.78
ASAT/ALAT	1.36 ± 0.11	1.24 ** ± 0.17	1.03 **^†^ ± 0.11
TAG (mmol/L)	3.07 ± 0.84	6.85 *** ± 1.16	4.02 ***^†^ ± 0.55
TC (mmol/L)	2.10± 0.36	2.72 *** ± 0.45	3.68 ***^†^ ± 0.31
HDL-C(mmol/L)	2.03 ± 0.27	1.72 *** ± 0.20	2.60 ***^†^ ± 0.22
LDL-C (mmol/L)	0.44 ± 0.12	0.96 *** ± 0.21	0.98 *** ± 0.20
HDL-C/LDL-C	2.65 ± 0.31	1.62 *** ± 0.39	2.08 ***^†^ ± 0.41
AIP	0.32 ± 0.08	0.51 *** ± 0.13	0.18 ***^†^ ± 0.05

AIP, atherogenic index of plasma; ALAT, alanine amino-transferase; ASAT, aspartate amino-transferase; CTRL, standard diet; HDL-C, high-density lipoprotein cholesterol; HF, high-fat diet; HF-Dia, high-fat diet supplemented with 12% of *D. lutheri*; HOMA-IR, homeostasis model assessment of insulin resistance; LDL-C, low-density lipoprotein cholesterol; TAG, triacylglycerol; TC, total cholesterol. Values are means (*n* = 6), with standard deviations represented by vertical bars. Statistical significance was determined using ANOVA with post hoc Fisher’s test and mean values were significantly different from those of the CTRL group with ** *p* < 0.01 or *** *p* < 0.001. ^†^ Significant difference compared with the HF group (*p* < 0.05).

**Table 2:** Effects of *D. lutheri* on inflammatory status.

Parameters	CTRL	HF	HF-Dia
**Plasma parameters**			
IL-6 (pg/mL)	26.83 ± 2.49	51.80 *** ± 7.83	56.87 ***^†^ ± 8.27
TNF-α (pg/mL)	22.13 ± 2.96	41.08 *** ± 3.30	42.11 *** ± 5.54
IL-4 (pg/mL)	41.11 ± 6.37	25.87 ***± 6.54	44.70 ***^†^ ± 6.32
**Adipocyte parameter**			
IL-10 (ng/mL)	0.73 ± 0.11	0.46 *** ± 0.08	0.73 ***^†^ ± 0.10

Effects of *D. lutheri* supplementation on inflammatory and anti-inflammatory biomarkers. The plasma concentrations of TNF-α, IL-6 and IL-4; IL-10 is the parameter measured in adipose tissue. CTRL, control group; HF, high-fat diet; HF-Dia, high-fat diet supplemented with 12% of *D. lutheri;* IL-4, interleukin-4; IL-6, interleukin-6; IL-10, interleukin-10; TNF-α, tumor necrosis factor-α. Values are means (*n* = 6), with standard deviations represented by vertical bars. Statistical significance was determined using ANOVA with post hoc Fisher’s test and mean values were significantly different from those of the CTRL group with *** *p* < 0.001. ^†^ Significant difference compared with the HF group (*p* < 0.05).

## Data Availability

The data presented in this study are all available in the present paper and supplementary materials.

## Supplementary Materials

The following are available online at https://www.mdpi.com/article/10.3390/molecules27134246/s1, Table S1: biochemical composition of standard, high-fat diets and freeze-dried *D. lutheri* biomass, Table S2: fatty acid composition of diets, Table S3: pigment and sterol composition, antioxidant activity and in vitro digestibility of freeze-dried *D. lutheri* biomass.

## Sample Availability

Samples of the compounds are not available.
