# Supplementary material for: The Potential of the Marine Microalga Diacronema lutheri in the Prevention of Obesity and Metabolic Syndrome in High-Fat-Fed Wistar Rats
Source: Molecules. 2022 Jun 30;27(13):4246. doi: 10.3390/molecules27134246 (PMC9268017; doi:10.3390/molecules27134246)
Supplement: Supplementary file 1 [file molecules-27-04246-s001.zip › molecules-1779092-supplementary.pdf]

Supplementary materials

Table S1. Biochemical composition of standard and high fat diets and freeze-dried *D. lutheri* biomass

	**CTRL***	**HF***	**Dia**	
Nutrients (g/100g)			Mean	SD
Proteins	16	19	35.4	0.64
Carbohydrates	60	32	13.46	0.48
Lipids	4	36	28.6	
Mineral and vitamin mixture	5	5	-	
DF	4	-	-	
Humidity	11	8	2.23	0.07
Ash	-	-	22.6	0.66

CTRL, standard diet; DF, dietary fiber; Dia, freeze-dried biomass of *D. lutheri*; HF, high fat diet; -, not determined. For CTRL diet, the mineral mixture provides the following amounts in mg/kg of diet: C, 7300; Cl, 4000; Cu, 16; Fe, 270; K, 6000; Mg, 1600; Mn, 70; Na, 2500; P, 5500; Zn, 55. The vitamin mixture of CTRL diet provides the following amounts in mg/kg of diet: biotin, 0.04; choline, 1600; folic acid, 0.5; niacin, 75; vitamin A, 7500; vitamin B1, 7; vitamin B2, 6.5; vitamin B5, 16.5; vitamin B6, 2.6; vitamin B12, 0.02; vitamin D3, 1000; vitamin E, 30; vitamin K3, 2.5. For HF diet the mineral mixture provides the following amounts in mg/kg of diet: Ca, 3000; Na, 4100; P, 5000. Results are represented as mean values ± SD, n = 3 for proteins, carbohydrates, humidity and ash of Dia biomass. * Data of biochemical composition of control and high fat diets have been furnished by SAFE.

Table S2. Fatty acid composition of diets

	CTRL (% molar)	HF (% molar)	Dia (g/100g)
Fatty acid	Mean	Mean	Mean
4:0	-	1.49	ND
8:0	-	1.20	ND
10:0	-	3.07	ND
12:0	-	3.95	ND
14:0	0.63	13.16	1.88
14:1	-	-	0.06
15:0	-	-	0.03
16:0	15.91	32.87	0.02
16:1n-7	0.73	-	1.48
16:2n-6	-	-	0.90
16:3n-3	-	-	ND
16:3n-6	-	-	ND
16:4n-1	-	-	ND
16:4n-6	-	-	ND
18:0	2.00	13.32	ND
18:1n-7	-	-	ND
18:1n-9	21.44	16.92	1.72
18:2n-6	49.84	5.44	0.5
18:3n-3	4.23	-	1.11
18:3n-6	-	-	0.05
18:4n-3	1.28	-	2.32
20:0	-	-	0.02
20:2n-6	-	-	0.03
20:3n-3	-	-	0.05
20:4n-3	-	-	ND
20:4 n-6	-	-	0.04
20:5 n-3	0.66	-	0.22
22:0	-	-	0.04
22:1	-	-	0.04
22:5n-3	0.91	-	ND
22:5n-6	-	-	0.19
22:6n-3	1.03	-	1.33
24:0	-	-	0.02
Others	1.24	8.58	0.02
Total SFA	18.54	69.06	3.58
Total MUFA	22.17	16.92	2.76
Total PUFA	58.05	5.44	5.85
Total n-3 PUFA	8.11	-	5.04
Total n-6 PUFA	49.94	5.46	0.82
n-6 PUFA/ n-3 PUFA	0.16	-	6.15
Total fatty acids	98.76	91.42	12.2

CTRL, standard diet; HF, high fat diet; ND, not detected; Dia, freeze-dried biomass of *D. lutheri*; -, not determined. Results are represented as mean values ± SD, n = 3 for Dia biomass. Mean values (n = 2) were reported, no statistical analysis were carried out, no SD mentioned.

Table S3. Pigment and sterol composition, antioxidant activity and *in vitro* digestibility of freeze-dried *D. lutheri biomass*

Pigments (g/100g)	Dia	
	Mean	SD
Total chlorophyll	1.82	0.88 10^{-1}
Chlorophyll-*a*	1.27	0.35 10^{-1}
Chlorophyll-*c*	0.54	0.52 10^{-1}
Total carotenoids	0.62	0.17 10^{-1}
Carotenoids (g/100g)		
Fucoxanthin	3.34 10^{-1}	
4k-hex-fucoxanthin	1.73 10^{-1}	
Dinoxanthin	4.00 10^{-2}	
β-carotene	1.50 10^{-2}	
Others	6.20 10^{-2}	
Sterols (g/100g)		
Cholesterol	8.37 10^{-3}	
Brassicasterol	7.00 10^{-1}	
24-Metilcholesterol	7.32 10^{-3}	
Campesterol	5.23 10^{-3}	
Stigmasterol	1.44 10^{-1}	
Fucosterol	1.78 10^{-1}	
Δ-5 Avenastérol	3.14 10^{-3}	
Total sterols	1.046	
Antioxidant activity*		
Antiradical activity DPPH (I_{50})† (mg biomass/ml extract)‡	0.33	
Antioxydant activity ORAC (µmol TE/mg biomass)§	27.4	
In vitro digestibility (% dry matter)	70.9 ± 3.08	

* Antioxidant activity was measured from extracts of Dia in 90% acetone. † I_{50}: concentration of the biomass/mL extract decreasing the absorbance of the DPPH solution by 50%. ‡ DPPH is a stable radical that can be reduced by reaction with an antiradical hydrogen-donor compound. § Trolox: water-soluble vitamin E analogue. 1 ORAC unit equals the net protection produced by 1 µM Trolox. DPPH, 2, 2-diphenyl-1-picrylhydrazyl; ORAC, Oxygen-radical absorbance capacity; TE, Trolox equivalent; Dia, freeze-dried of *D. lutheri*. Results are represented as mean values ± SD (not reported), n = 3, excepted for sterol composition (n = 1).
